# Antibiofilm, Anti-Inflammatory, and Regenerative Properties of a New Stable Ozone-Gel Formulation

**DOI:** 10.3390/pharmaceutics16121580

**Published:** 2024-12-11

**Authors:** Carla Russo, Giuseppe Curcio, Alessandro Graziani, Antonella Mencacci, Donatella Pietrella

**Affiliations:** 1 Medical Microbiology Unit, Department of Medicine and Surgery, University of Perugia, Piazzale Severi, Building D, 4th Floor, 06129 Perugia, Italy; carla.russo179@dottorandi.unipg.it (C.R.); curcio.g.1995@collaboratori.unipg.it (G.C.); alessandro.graziani@dottorandi.unipg.it (A.G.); antonella.mencacci@unipg.it (A.M.); 2 Microbiology Unit, Santa Maria Della Misericordia Hospital, 06129 Perugia, Italy

**Keywords:** ozone, biofilm, antimicrobial activity, multi-drug resistant microorganisms, regenerative activity, anti-inflammatory activity

## Abstract

**Background/Objectives**: Chronic skin wounds are characterized by inflammation, persistent infections, and tissue necrosis. The presence of bacterial biofilms prolongs the inflammatory response and delays healing. Ozone is a potent antimicrobial molecule, and many formulations have been used in the advanced therapeutic treatment of chronic wounds. The aim of this work was to determine the antimicrobial, anti-inflammatory, and regenerative activity of a stable ozone-gel formulation over time. **Methods**: The antimicrobial property was assessed by measuring the minimal inhibitory concentration and the antibiofilm activity. The anti-inflammatory effect was evaluated by TNF-α determination, and the regenerative effect was measured by scratch assay. **Results**: The ozone gel demonstrated antimicrobial and antibiofilm activity in all ATCC microorganisms examined and on most clinical isolates. Higher concentrations of the ozone gel were also useful in the dispersion of preformed biofilm. The ozone gel also showed anti-inflammatory activity by reducing the production of TNF-α and regenerative activity in human fibroblasts and keratinocytes. **Conclusions**: Given all these antimicrobial, anti-inflammatory, and regenerative characteristics, the ozone gel could be, in this formulation, used in the treatment of wounds. The ozone-gel formulation described here retains stability for over 30 months, which facilitates its use compared to formulations that lose efficacy quickly.

## 1. Introduction

Skin wounds are very frequent and have a remarkable impact on healthcare systems burdening the countries’ care system. Skin wounds can be classified into acute and chronic on the base of pathogenesis. Acute wounds undergo healing and recovery of structural integrity. Conversely, chronic wounds are distinguished by inflammation, persistent infections, and tissue necrosis. Additionally, the presence of bacterial biofilms prolongs the inflammatory response. This condition predisposes to difficult wound healing [1] or non-healing wounds, generally associated with comorbidities such as aging, hypertension, diabetes, and vascular deficits (primarily venous ulcers, pressure sores, and diabetic foot ulcers) [2]. Current treatments are based on euglycemia, antimicrobials, debridement, and dressing applications [3]. Alternative natural and plant-based products, such as honey, aloe vera, oils, and calendula, are used for wound healing therapies.

Pathogens that commonly infect wounds include *Staphylococcus aureus* and *Pseudomonas aeruginosa*, which express virulence factors for adherence and invasion. Polymicrobial biofilm formation contributes to antibiotic resistance. Anaerobic organisms, fungi, and viruses as well as drug-resistant microorganisms pose a challenge to diagnosis and therapy [4]. *P. aeruginosa*, *Klebsiella pneumoniae*, *Acinetobacter baumanni*, *Enterobacter* spp., *Proteus* spp., and *Escherichia coli* are the most commonly isolated Gram-negative bacteria from burn wounds [5]. Reduced microcirculation in the diabetic foot and impaired immune response and infections by multidrug-resistant and biofilm-forming microbes contribute to the development of chronic, non-healing wounds [6].

Ozone (O_3_) is considered a powerful antimicrobial molecule, and it is used in advanced therapeutic treatment of chronic wounds with better healing outcomes. Ozone causes irreversible injury to microbial DNA [7] and membrane phospholipids [8,9]. Moreover, the Reactive Oxygen Species (ROS) produced by decomposed O_3_, such as superoxide anion radical (O_2_^−^), hydroxyl radical (^.^OH), and nitric oxide (^.^NO), act as vasodilators and stimulators of growth factors [9,10]. However, high levels of ROS can be toxic, and concentration must be monitored [11]. Low doses of ozone are able to trigger several useful biochemical mechanisms and reactivate the antioxidant system [12]. Ozone is frequently used as a complementary therapy for cutaneous and skin diseases (eczema, dermatitis, psoriasis), wound healing, diabetic foot, and pressure ulcers but disadvantages associated with ozone are its low solubility and instability when dissolved in an aqueous solution [13]. Ozone can be formulated for therapy in water or oil and administered as autohemotherapy [14]. A thermoresponsive ozone-enriched spray gel has been developed to inhibit the tumor recurrence of hepatocellular carcinoma thanks to its property of tumor cells killing by generating reactive oxygen species [15]. An innovative hydrogel based on hyaluronic acid containing micro/nanocapsules of ozonated olive oil has shown interesting regenerative properties and an inhibitory effect against skin microbiota and pathogenic yeasts [16]. A previous study has shown that the use of an ozone-based gel provides several benefits in the healing of post-extraction dental wounds. In evaluating parameters related to post-operative recovery, a faster reduction in perceived pain was observed in treated subjects compared to the control group. Additionally, subjects treated with the ozone-based gel showed greater mouth-opening ability by the fifth day compared to those treated with a saline solution. The control group received saline irrigation and a 5-day course of antibiotics and painkillers, while the study group had the socket filled with ozone gel, applied three times daily for 5 days, without antibiotics [17]. This highlights one of the main advantages of using ozone gel: it replaces antibiotic treatments, thereby reducing the risk of bacterial resistance and preserving the endogenous microbiota. Moreover, the effective use of ozonized gel in treating periodontal disease has been demonstrated, comparing the outcomes with those of the therapeutic gold standard (nonsurgical treatment in combination with chlorhexidine gel). The use of ozonized gel yielded outcomes nearly identical to the gold standard, offering an important advantage of maintaining a broad spectrum of action with reduced toxicity with respect to chlorhexidine [18]. Furthermore, chlorhexidine has been reported to cause severe allergic reactions, such as anaphylactic shock, urticaria, and even cardiac arrest [19]. Ozone as an active ingredient can be prepared in various formulations: oxygen/ozone gas, ozonated oil/gel, and ozonated water [20]. Although the properties of ozone are similar in the various formulations, what substantially varies is the level of stability and release. Gaseous ozone is both toxic and unstable, while ozonated water tends to break down more quickly than gels [21]. Using ozone in an oil-based gel form may offer greater stability, allowing it to remain effective on the target surface for a longer duration [22]. One of the main advantages of using an ozone-gel formulation is the ability to apply the compound directly to the affected area, allowing for direct and more prolonged contact with the injured site.

The aim of the present research was to study an innovative hydrogel based on stable ozonized glycerin and to assess its antimicrobial, anti-inflammatory, and regenerative tissue properties.

## 2. Materials and Methods

### 2.1. Ozone Gel

The ozone gel was produced and supplied by Bioxid AD (Forte dei Marmi, Lucca, Italy). The present ozone gel was produced specifically for this study. Ozone gel contains water, ozonized glycerin, carbomer, triethanolamine, propylene glycol, imidazolidinyl urea, potassium sorbate, and disodium EDTA. The control gel used in experiments contains all the components except ozonized glycerin. The gels are prepared with a hot turbo mixer. Ozone gel was produced by the following process: a system passed distilled water through a micronizing filter that mixed ozone into the water. The water, propelled by a high-pressure transfer pump, passed through a ceramic filter that broke down the ozone, generating ionized oxygen. The water was immediately gelled. In the gelation process, approximately 70/75 percent of ions were lost. The final concentration of ozone in the gel was 0.02%. After 30 months, around 20% was lost. The stability of ozone in the gel was evaluated by a specific multiparametric probe (Hanna Instruments, Padova, Italy). The release during application was immediate. Ozone gel and control gel were provided by Munus International Srl (Perugia, Italy).

### 2.2. Microorganisms

The microbial strains used in this study were two Gram-positive bacteria, *Staphylococcus aureus* (ATCC 25923) and *Staphylococcus epidermidis* (ATCC 35984), the Gram-negative bacteria *Pseudomonas aeruginosa* (ATCC PAO-1) and *Escherichia coli* (ATCC 86963), and the yeast *Candida albicans* (strain SC5314/ATCC MYA-2876). Bacterial cultures were maintained in Mueller Hinton Agar (MHA). *Candida albicans* was maintained in Sabouraud agar (SAB). The day before the test, one colony was inoculated in 7 mL of Mueller Hinton broth (MHB) or SAB broth and incubated for 24 h at 37 °C. In addition, 58 clinical isolates were obtained from the Microbiology Unit of the Santa Maria della Misericordia Hospital in Perugia, Italy: *E. coli*, ESBL *E. coli*, *Klebsiella pneumoniae* resistant to carbapenems (KPC), four *Candida* species (*C. glabrata*, *C. albicans*, *C. kefir*, *C. parapsilosis*), *Streptococcus dysgalactiae*, *Enterococcus faecalis*, *Enterococcus faecium*, *S. aureus* methicillin resistant (MRSA), and *Pseudomonas aeruginosa*.

### 2.3. Minimal Inhibitory Concentration (MIC) Assay

The Minimal Inhibitory Concentration (MIC) was determined by the microbroth dilution method according to the Clinical and Laboratory Standards Institute/National Committee for Clinical Laboratory Standards (CLSI/NCCLS) Approved Standard M100-S21, 2007). Gentamicin and fluconazole solutions were used as positive controls for bacteria and fungi, respectively. Ozone-gel dilutions were prepared in MHB; the dilutions, ranging from 500 to 3.9 μL/mL, were prepared in U-bottom 96-well plates. Overnight microbial cultures were then diluted in sterile saline, and the concentration of cells was determined by measuring optical densities at 600 nm using a spectrophotometer (Infinite M200 pro, TECAN, Männedorf, Switzerland). The inoculum size of bacteria was 1–2 × 10^4^/mL. In each well, 100 μL of microbial suspension was added. Plates were then incubated at 37 °C for 24 h. The MIC of ozone gel was defined as the lowest concentration that inhibited visible growth of the microorganisms.

### 2.4. Ozone-Gel Effect on Biofilm Formation

The in vitro static biofilm assay was performed using a 96-well flat-bottom microtiter plate as previously described with some modifications [23]. To grow biofilms, overnight cultures of bacteria were diluted 1:100 into 15 mL of MHB supplemented with 2% sucrose, in the presence or absence of ozone gel at the concentrations indicated (250, 125, and 62.5 μL/mL) or control gel. Cultures were incubated at 37 °C for 24 h under static conditions. After incubation, the biofilm developed in each well was washed twice with 200 μL of distilled water, and then, biofilm bacteria were resuspended in saline, and their concentration was determined by measuring optical densities (OD) at 600 nm using a spectrophotometer (Infinite M200 pro, TECAN, Männedorf, Switzerland). Biofilm formation bioassays were performed in triplicate for at least three independent experiments at each concentration.

### 2.5. Effect of Ozone Gel on Biofilm Dispersion

Biofilms were grown in a 96-well flat-bottom microtiter plate as described above. Preformed biofilms were treated with three different concentrations of ozone gel (250, 125, and 62.5 μL/mL) as dispersion inducers or just the control gel, and incubated at 37 °C for a further 24 h. The positive control for Gram-positive and Gram-negative bacteria was gentamicin, and fluconazole for *C. albicans*. Afterward, the biofilm was quantified by measuring OD at 600 nm. Biofilm dispersal bioassays were performed in triplicate for at least three independent experiments at each concentration. Results are reported as a percentage of biofilm dispersion through the following formula:

Biofilm dispersion = 100 − (OD ozone-gel-treated preformed biofilm ×  100)/OD control-gel-treated preformed biofilm

### 2.6. Peripheral Human Mononuclear Cells (PBMC) Isolation

Heparinized venous blood was obtained from a buffy coat gently provided by the Blood Bank of the Ospedale della Misericordia of Perugia. All donors have been informed, and they signed the consensus form (MOSIT 06) approved by the Ethics Committee CEAS (Comitato Etico Aziende Sanitarie) (Rev. 3 Ottobre 2014), in which they authorize the use of their samples for research studies. Human peripheral blood mononuclear cells (PBMC) were separated by density gradient centrifugation over Ficoll-Hypaque Plus (Pharmacia Biotech), recovered, washed twice, and suspended in RPMI 1640 supplemented with 10% FBS, 100 U penicillin/mL, and 100 µg streptomycin/mL.

### 2.7. Cytotoxicity Assay

The cytotoxicity was tested by the determination of the cell ATP level by ViaLightPlus Kit (Lonza). The method is based on the bioluminescent measurement of ATP, which is present in all metabolically active cells. The bioluminescent method utilizes luciferase, an enzyme that catalyzes the formation of light from ATP and luciferin. The emitted light intensity is linearly related to the ATP concentration, and it is measured using a luminometer. To perform cytotoxicity tests, PBMC were recovered, counted, and adjusted to the concentration of 2 × 10^5^/mL, seeded in a flat-bottom 96-well culture plate, and incubated in the presence of scalar dilutions of ozone gel (250, 125, 62.5, 31.125, 15.6, 7.8, and 3.9 µL/mL) prepared in cRPMI. Each concentration was tested in triplicate. After adding ozone gel to PBMC, plates were incubated for 4 and 24 h at 37 °C. Ozone gel was tested on human dermis fibroblasts (HuDe) and human skin keratinocytes (NCTC2544) cells, which were grown in cRPMI overnight to reach confluence. Monolayer cells were treated for 24 h at 37 °C with scalar dilutions of ozone gel (250, 125, 62.5, 31.125, 15.6, 7.8, and 3.9 µL/mL) prepared in cRPMI. After incubation, plates were left to cool at room temperature for 10 min, and then the Cell Lysis Reagent was added to each well to extract ATP from cells. Next, the AMR Plus (ATP Monitoring Reagent Plus) was added, and after 2 min, the luminescence was read using a microplate luminometer (TECAN). Results are expressed as the 50% cytotoxic concentration (CC50), the concentration required to reduce cell viability by 50% compared to the untreated controls.

### 2.8. Cytokine Determination

PBMC were pre-stimulated with lipopolysaccharide (LPS 1 µg/mL, Merk Life Science S.r.l., Milano, Italy) for 4 h, and then ozone gel 6 µL/mL was added to the cultures for a further 24 h; in parallel experiments, PBMC were costimulated with LPS and ozone gel for 24 h. Culture supernatants were recovered and stored at −20 °C until cytokine determination. Secreted TNF-α was determined using an immune assay, ELISA (U-Cytech Biosciences, Utrecht, The Netherlands).

### 2.9. Scratch Assay

HuDe fibroblasts or NCTC2544 keratinocytes were seeded into each well of a 96-well plate and allowed to grow in cRPMI until a 90% confluent monolayer was achieved. Then, a scratch was made in the center of the cell monolayer with a sterile pipette tip, and the plates were washed several times with PBS to remove cellular debris. At this point, PBS was replaced with cRPMI containing 5–10 µL/mL ozone gel or control gel for 72 h. Digital images were obtained to quantify the initial scratch width (0 h) using an inverted microscope. To quantify scratch closure rates, digital images were obtained at 0 and 24, 48, or 72 h, as indicated. The extent of wound closure was determined by measuring the gap between cells at five different points. Results were reported as the mean ± SD.

### 2.10. Statistical Analysis

Differences between ozone-gel-treated biofilm and untreated biofilm or between human PBMC, HuDe, or NCTC2544 treated with ozone gel and untreated cells were compared using the Student’s *t*-test (two-tailed) with Excel software. * *p*-values of <0.05 were considered significant.

## 3. Results

### 3.1. Ozone-Gel Stability

Ozone gel was stored at room temperature, and its stability was evaluated every 2 months. The ozone concentration remained stable for up to 24 months; during the next 6 months, a reduction of ozone up to 5–20% was observed.

### 3.2. Antimicrobial Activity

The antimicrobial activity of ozone is already known. Several formulations have been described and tested in orthopedic settings [24], oral cavity infections [18], and diabetic foot ulcers [25]. In order to use ozone gel in wound treatment, the ozone gel has been tested on microorganisms generally involved in wound infections, such as *S. aureus*, *Staphylococcus epidermidis*, *P. aeruginosa*, *E. coli*, and yeasts such as *Candida albicans*. The ozone-gel formulation was first tested for its antimicrobial properties and then for its activity on biofilm formation and dispersion.

The inhibitory activity of the microorganism growth was determined by MIC assay. In the first experimental series, the antimicrobial activity of the ozone gel was tested on ATCC strains of *S. aureus* (ATCC 25923), *S. epidermidis* (ATCC 35984), *P. aeruginosa* (ATCC PAO-1), *E. coli* (ATCC 86963), and the yeast *C. albicans* (ATCC CAF2-1).

The ozone gel was tested at 1:2 serial dilutions, and the MIC was considered the higher dilution in which no microbial growth was detected. As a positive control, the antibiotic gentamicin or fluconazole for bacteria and yeast, respectively, were used. As reported in Table 1, ozone gel was active against *C. albicans* at 62.5 μL/mL, *S. aureus* at 125 μL/mL, and *S. epidermidis*, *P. aeruginosa*, and *E. coli* at 250 μL/mL. Ozone-free gel was used as a negative control in all experiments. In the absence of ozone, no antimicrobial activity was detected.

The antibiofilm activity of ozone gel was evaluated at 500, 250, and 125 μL/mL. The biofilm formation of the Gram-positive bacteria *S. aureus* ATCC 25923 and *S. epidermidis* ATCC 35984 was reduced up to 250 μL/mL and 125 μL/mL, respectively (Figures S1A and S2A), while only 500 μL/mL was able to break down the biofilm formed after 24 h of incubation (Figures S1B and S2B). The results obtained for ozone gel at the concentration of 500 μL/mL for *S. aureus* are comparable to those obtained with treatment with gentamicin used at the concentration of 20 μg/mL. Conversely, for *S. epidermidis*, gentamicin was not able to reduce significantly the mass of the preformed biofilm; however, a dispersion of the biofilm with ozone gel at 500 μL/mL was observed.

The biofilm formation of the Gram-negative bacteria *E. coli* ATCC 86963 and *P. aeruginosa* ATCC PAO-1 was reduced up to 125 μL/mL (Figures S3A and S4A), but only the concentration of 500 μL/mL was able to break down the *E. coli* biofilm but was not able to reduce the mass of the preformed *P. aeruginosa* biofilm obtained after 24 h of incubation (Figures S3B and S4B). The results obtained at the concentration of 500 μL/mL of ozone gel are comparable to those obtained with gentamicin treatment.

Similarly to the results obtained for the Gram-positive bacterium *S. epidermidis*, the biofilm formation of the yeast *C. albicans* was reduced by all ozone-gel concentrations used (Figure S5A), as observed for fluconazole. However, for dispersion, fluconazole was not able to reduce the preformed biofilm, whereas ozone gel was capable of reducing the mass of the biofilm at the concentration of 500 μL/mL (Figure S5B).

Given the antimicrobial activity of ozone gel in the first series of experiments carried out on ATCC strains, the antimicrobial activity of ozone gel was tested on 58 clinical isolates: *E. coli*, extended-spectrum β-lactamases (ESBLs) producing *E. coli*, carbapenemases producing *K. pneumoniae* (KPC), several *Candida* species *(C. glabrata*, *C. albicans*, *C. kefir*, *C. parapsilosis)*, *Streptococcus dysgalactiae*, *Enterococcus faecalis*, *Enterococcus faecium*, Meticillin-resistant *S. aureus* (MRSA), and *P. aeruginosa*. Gentamicin and Fluconazole were used as positive controls for bacteria and yeasts, respectively. The antibiofilm activity was evaluated at concentrations 250, 125, and 62.5 µL/mL. As negative control, ozone-free gel was used; no anti-biofilm activity was detected.

The growth of all clinical Gram-negative isolates was inhibited at 250 or 125 µL/mL.

An appreciable inhibition of biofilm formation was observed up to 81% at 250 µL/mL. The ozone gel at 125 µL/mL reduced the biofilm formation by 69% to 75%. Conversely, the dispersion of the preformed biofilm with 250 µL/mL of ozone gel varied among the different clinical isolates, ranging from 36% to 51% (Table S1).

ESBLs *E. coli* produces β-lactamases that hydrolyze a wide variety of β-lactam antibiotics, including penicillins, cephalosporins, and monobactams, making them multi-resistant microorganisms. A significant inhibition biofilm formation in all clinical isolates at 125–250 µL/mL of ozone gel (Table S1) was observed. 250 µL/mL of ozone gel also managed to disperse the biofilm by up to 73%.

*K. pneumoniae* is an opportunistic pathogen that typically colonizes the human gastrointestinal tract, skin, and upper respiratory tract. Most infections caused by *K. pneumoniae* are hospital-acquired and mainly include respiratory tract infections and bacteremias associated with high mortality. Over time, *K. pneumoniae* has developed resistance to several classes of antibiotics. Recently, the majority of *K. pneumoniae* infections have been caused by the KPC isolates, i.e., the bacterium produces carbapenemases that inactivate β-lactams. Both concentrations of 250 and 125 µL/mL led to a high reduction in biofilm formation in all clinical isolates up to 89% (Table S1). Dispersion of the preformed biofilm was observed after treatment with both 250 and 125 µL/mL of ozone gel.

*Pseudomonas aeruginosa* is a common environmental Gram-negative organism; it can be a significant pathogen of serious opportunistic infections in humans. Generally, it infects the airways and urinary tracts, causes blood infections, and is the most common cause of burn infections and dermatitis. Furthermore, it is the most frequent colonizer of medical devices and is one of the pathogens that cause nosocomial infections, such as pneumonia. Treatment of *P. aeruginosa* infections can be difficult due to its natural and acquired resistance to antibiotics [26]. Our experimental data obtained by six *P. aeruginosa* clinical isolates demonstrated that ozone gel (250 and 125 µL/mL) reduced biofilm in most clinical isolates by 37–82% (Table S1). However, its effect on the dispersion of the biofilm was not relevant.

In the second series of experiments, we evaluated the antimicrobial activity of ozone gel against Gram-positive bacteria. The growth of all clinical isolates was inhibited at 250 or 125 µL/mL.

*S. aureus* is a coagulase-positive Gram-positive pathogen belonging to the *Staphylococcaceae* family. *S. aureus* is one of the main human pathogens, presenting many virulence factors and also a notable ability to acquire resistance to most antibiotics, as in the case of MRSA. In most cases, MRSA accounts for 25–50% of *S. aureus* infections in hospital settings [27]. The percentage of inhibition of biofilm mass is significant at 250 and 125 µL/mL, reaching values up to 90%. Dispersion of the biofilm was observed only for some clinical isolates after treatment with 250 µL/mL of ozone gel, with a maximum reduction of 63%.

*Streptococcus dysgalactiae* is a Gram-positive, beta-hemolytic bacterium belonging to the *Streptococcaceae* family. These bacteria frequently cause throat, skin, and soft tissue infections and can spread widely to many deep tissue sites, including the endocardium.

Two clinical isolates presented a reduction of biofilm formation in the presence of ozone gel at 250 µL/mL of about 80%, while only one clinical isolate showed a reduction of the biofilm at 125 µL/mL (Table S2). Dispersion of the biofilm was not observed for two isolates, with a significant reduction of 90%.

*Enterococcus faecalis* is a Gram-positive coccus belonging to the *Enterococcus* genus. It appears to be one of the most frequently isolated bacterial species in all types of wounds, including diabetic foot ulcers, burns, and surgical sites. *E. faecalis* infections are increasingly difficult to treat due to their intrinsic and acquired resistance to a variety of antibiotics. The bacterium initially adheres and colonizes and subsequently must overcome the host’s defenses to stabilize the infection. Biofilm formation, together with the expression of substrate aggregation substance, can promote the survival of *E. faecalis* within macrophages and neutrophils [28]. 250 µL/mL and 125 µL/mL of ozone gel lead to a reduction of the biofilm of the first and third isolates, while for the second one, the reduction of biofilm occurs only at the higher concentration. The dispersive effect of the ozone gel on the preformed biofilm was highlighted only for the first and third clinical isolates when treated with 250 µL/mL of ozone gel.

*Enterococcus faecium* belongs to the normal bacterial microbiota of the gastrointestinal tract. It is considered harmless commensals in healthy subjects, but under certain conditions, it can cause endocarditis, sepsis, and urinary tract infections, and it has been associated with peritonitis and intra-abdominal abscesses in the hospital environment.

Reduction of biofilm formation at 250 µL/mL was between 64% and 74%. However, at 125 µL/mL, the decrease in biofilm mass was not significant for any isolates (Table S2). No dispersion was detected with either ozone-gel concentration.

*Candida* infections have increased considerably over the last three decades. The genus *Candida* contains over 150 heterogeneous species, but only a minority have been implicated in human candidiasis. Among *Candida* species, *C. albicans* is the main cause of candidiasis, but other species have also been identified as human pathogens. The pathogenicity of *Candida* is facilitated by a series of virulence factors, such as adhesion to host surfaces, medical devices, biofilm formation, and secretion of hydrolytic enzymes [29]. Furthermore, *Candida* can colonize burn wounds, especially the *C. parapsilosis* species, and cause the development of candidemia [30].

In our experimental model, the growth of all clinical *Candida* isolates was inhibited at 125 or 62.5 µL/mL (Table S3). For all isolates of *C. albicans*, the reduction in biofilm formation was significant, up to 90% at concentrations of 250, 125, and 62.5 µL/mL. This data confirms previous observations for *C. albicans* ATCC MYA-2876. The dispersive effect on the biofilm was confirmed only in the last two clinical isolates, with percentages in a range of 68–74% for ozone gel at 250 µL/mL and about 40% at 125 µL/mL.

*Candida glabrata* biofilm formation was inhibited by all ozone-gel concentrations tested, while no biofilm dispersion was observed.

A single clinical isolate of *Candida kefir* and *C. parapsilosis* was analyzed. Antibiofilm activity was observed up to approximately 75% at the three concentrations tested. *C. kefir* biofilm dispersal was also observed after treatment with ozone gel at 250 and 125 µL/mL, the results were up to 81% and 59%, respectively (Table S3). A reduction in biofilm formation and dispersion was detected after treatment with 250 and 125 µL/mL of ozone gel.

### 3.3. Cytotoxic and Anti-Inflammatory Activity

**Figure 1:**
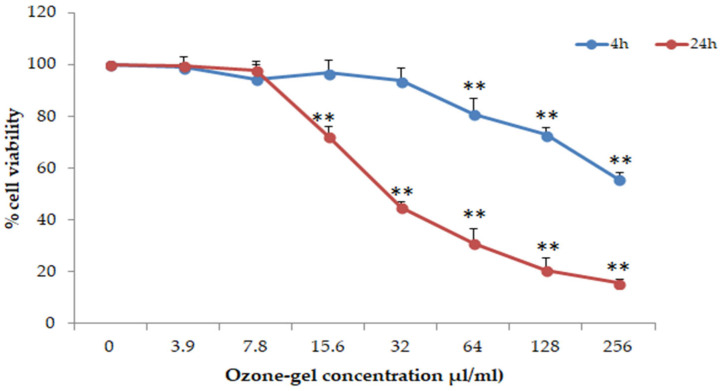
Cytotoxicity of ozone gel on human PBMC. Results are expressed as the percentage of live cells with respect to untreated cells, assumed to be 100. The results are expressed as mean ± standard deviation (SD) of two independent experiments conducted in triplicate. The statistical analysis was performed with a two-tailed Student’s *t*-test. ** *p* < 0.01, ozone-gel-treated cells vs. untreated cells.

The anti-inflammatory activity of ozone gel was tested on ex vivo human PBMCs from healthy donors. First of all, the cytotoxic effect of the ozone gel at 4 and 24 h was determined through the ViaLightPlus assay, which quantifies the ATP produced by live cells. The CC_50_ evaluated after 4 and 24 h of incubation was 250 and 31.25 µL/mL, respectively. As shown in Figure 1, at 4 h, the percentage of live cells remains around 100%, up to 31.25 µL/mL. At increasing concentrations, the percentage of live cells progressively decreases. After 24 h of incubation, 100% live cells are observed only up to a concentration of 7.8 µL/mL. The percentage of live cells dropped to 20% after treatment with 250 or 125 µL/mL of ozone gel.

**Figure 2:**
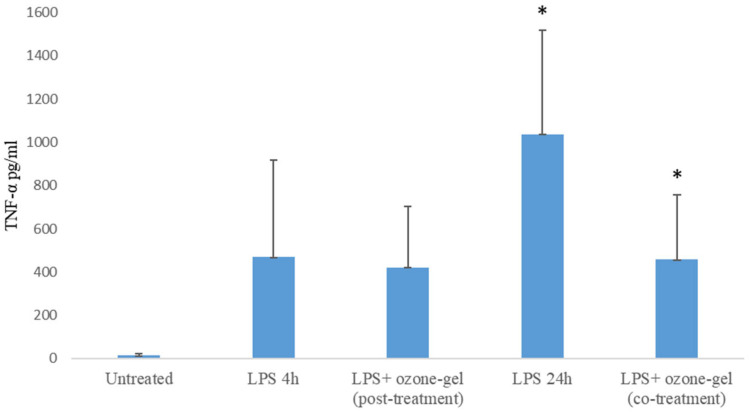
Effect of ozone gel on TNF-α production by human PBMC. Cells were pre-stimulated with LPS and then treated with ozone gel or were co-stimulated. Data represent the mean ± SD of three independent experiments. The statistical analysis was performed with a two-tailed Student’s *t*-test. * *p* < 0.05, LPS+ozone-gel-treated cells vs. LPS-treated cells.

Based on these results, since the anti-inflammatory activity was performed at 24 h, the concentration of 6 µL/mL was chosen. The cells were stimulated with the inflammatory bacterial component (lipolysaccharide, LPS) and ozone gel. LPS-stimulated PBMCs produce TNF-α compared to untreated PBMCs, at 4 and 24 h of treatment. Co-treatment of PBMCs with LPS and ozone gel causes a significant reduction (*p* = 0.03) in cytokine production compared to PBMCs stimulated with LPS alone (Figure 2). However, post-treatment with ozone gel after a 4-h stimulation of cells with LPS did not significantly reduce TNF-α production (*p* = 0.74).

### 3.4. Regenerative Activity of Ozone Gel

**Figure 3:**
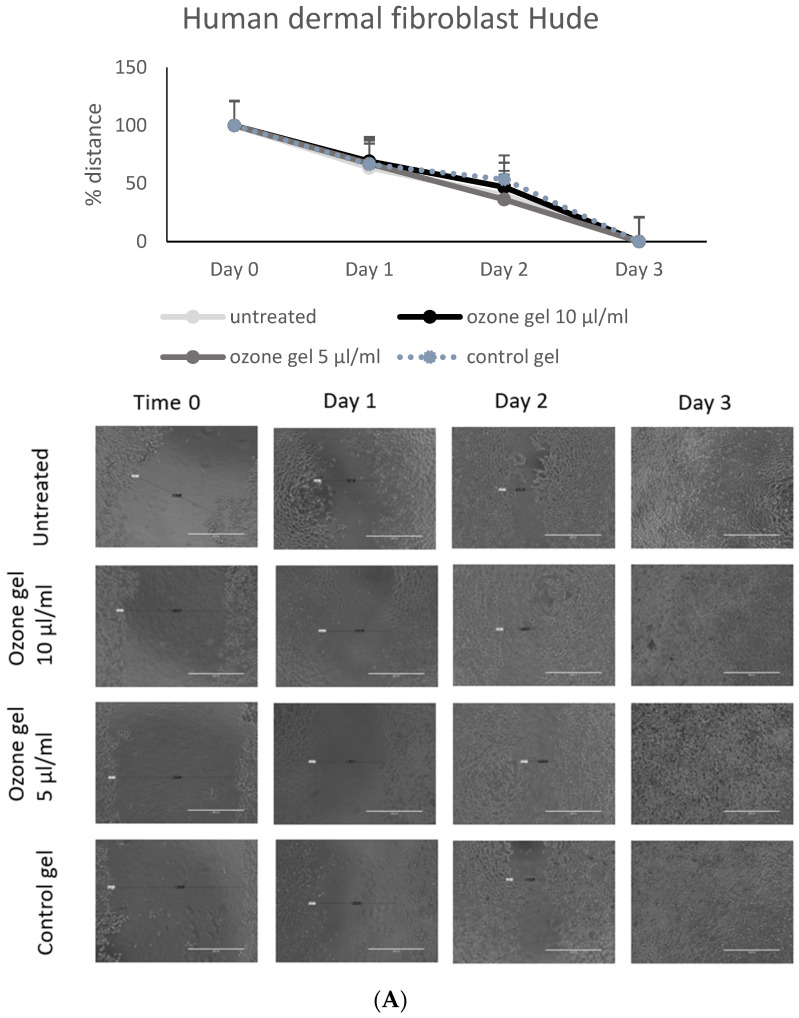

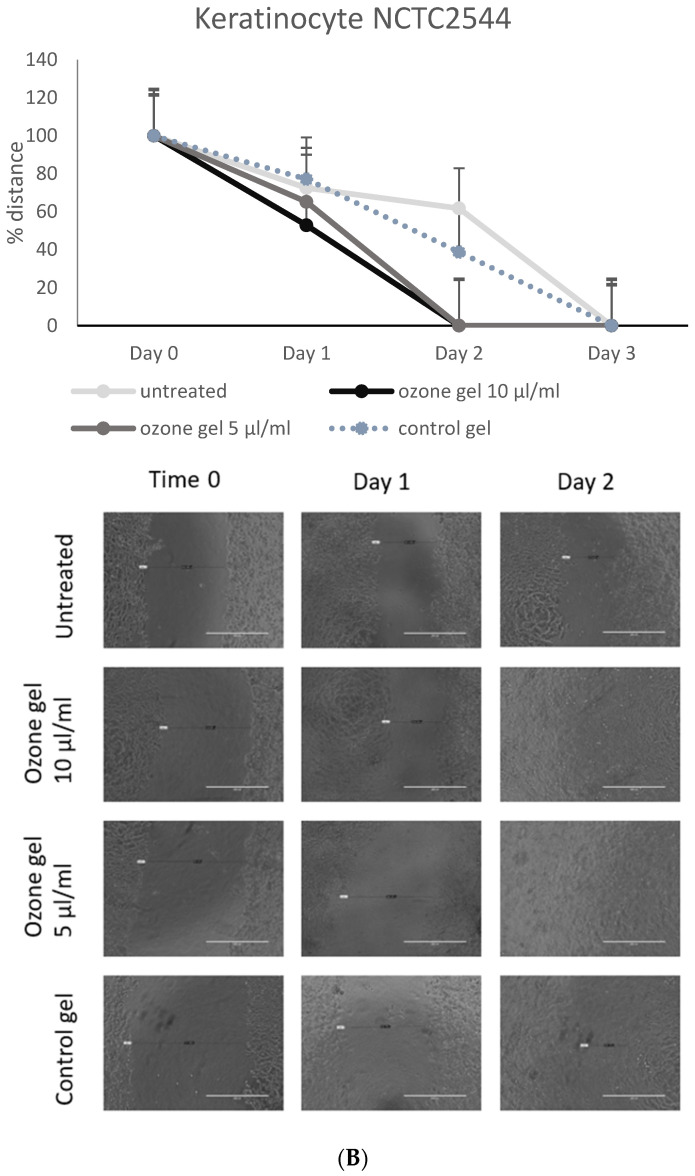
Regenerative effect of ozone gel on human dermal fibroblasts (**A**) and keratinocytes (**B**). The regenerative activity was performed by a scratch assay. The distance between the edges of the scratch was monitored under the microscope (20× magnification) for 2 or 3 days as indicated, and photos were taken. The distances were recorded, and the results are expressed as mean ± SD of the measurements by microscope (n = 6) carried out in two independent experiments.

Wound healing is often affected by the inflammatory process and infections due to the formation of microbial biofilms and the inability of skin cells to respond to regenerative stimuli. Cytotoxicity of ozone gel was tested on human dermal fibroblast (HuDe) and human keratinocyte (NCTC2544) cell lines. The concentration of 5 and 10 µL/mL did not affect the viability of either cell line (CC_50_ was 139 and 84.6 µL/mL for HuDe and NCTC, respectively). The regenerative capacity of the ozone gel on HuDe was evaluated using the scratch test, which allows to evaluate the proliferation of dermal cells during the closure of the spaces (Figure 3A) in the presence of 5–10 µL/mL of ozone gel or control gel. Cell distances were monitored under a microscope and measured for 3 days. The control gel had no effect on cell regeneration. The results showed that both concentrations used induced better regenerative activity than the control gel. The effect is already evident after 2 days, and by the third day, all scratches were closed. Both concentrations of ozone gel used accelerated frame closure of keratinocytes by 24 h compared to the control gel and untreated cells (Figure 3B).

## 4. Discussion

Ozone has been identified as a potential chemical agent capable of inactivating, at sufficient concentrations, various bacterial species. Bacteria are highly susceptible to oxidative stress. Increased levels of ROS are known to damage iron–sulfur clusters, proteins, and DNA [31]. Reactive species formed on proteins that are exposed to singlet oxygen, [32]; protein oxidation can generate derivatives susceptible to proteolytic degradation, alter amino acid residues, and lead to the cleavage of polypeptide chains [33]. Proteins affected by senescence-related oxidation include several enzymes involved in essential bacterial processes, such as the DnaK chaperone from the Hsp70 family, histone-like proteins, translation elongation factors, the universal stress protein A, and enzymes of the Krebs cycle [34]. The data reported in this work on the antimicrobial activity of ozone gel on *S. aureus* confirm what has already been described in the literature for other formulations. Ozonated water is able to inhibit the growth of several isolates of *S. aureus* [35]. Further evidence of the antimicrobial activity of ozone against *S. aureus* is provided by a study conducted on individuals suffering from atopic dermatitis. In this study, it was observed that in patients treated with ozone therapy administered topically, the percentage of *S. aureus* decreased after a three-day treatment. Furthermore, the diversity of the skin microbiota was restored in lesions caused by atopic dermatitis [36]. Similarly to the findings for *S. aureus*, the data on the antimicrobial activity of ozone gel on *S. epidemidis* confirm those reported in the literature for other formulations. Two of the formulations studied involve the use of wearable biomedical systems or patches capable of releasing ozone locally, allowing a continuous and long-term treatment regime at low doses. Through these ozone-releasing systems, a reduction exceeding 70% compared to the initial inoculation of *S. epidermidis* was observed during 6 h of exposure to ozone [37]. Other studies on the use of ozone generated via a syringe for application to the infected tooth root demonstrated the action of ozone on three different bacteria. The method was not 100% effective but led to a significant decrease in surviving microorganisms. In particular, for *S. epidermidis*, an efficacy rate of 88.6% was recorded [38].

The activity of ozone on *E. coli* is also confirmed by studies reported in the literature, which state that it is possible to reduce the concentration of *E. coli* in hospital wastewater after treatment with gaseous ozone. The reduction depends on the dose of ozone used and the contact time [39]. Other studies, however, demonstrated that exposure to low concentrations of gaseous ozone on suspensions of various bacteria, such as *A. baumannii*, *S. aureus*, *Salmonella enterica*, *P. aeruginosa*, and *E. coli*, did not significantly reduce the growth of bacteria compared to control groups. However, a significant reduction in the metabolic activity of *E. coli* was observed [9]. Ozonated water is mainly used in the disinfection of medical instruments, but it has been hypothesized that it could also be used as a hand sanitizer [40]. The antimicrobial activity of ozone produced using vacuum UV lamps on some multi-resistant bacteria, including ESBL *E. coli*, has been observed [41].

The antimicrobial activity of ozone gel against *P. aeruginosa* is comparable to that observed in the literature. As previously mentioned, patches or wearable biomedical systems capable of locally releasing ozone onto wounds were studied. These devices, in addition to being effective on *S. epidermidis*, are capable of acting against *P. aeruginosa*. Notably, it has been demonstrated that the activity of ozone thus formulated on *P. aeruginosa* is even better than on *S. epidermidis*. This conclusion is supported by evidence that the treatment completely eradicates the preformed biofilm in 6 h [23]. Even ozonated air can have a bactericidal effect against *P. aeruginosa*. Experimental data have correlated a better antimicrobial activity with an increase in the ozone dosage and contact time; the treatment causes the rapid release of K^+^, Mg^++^, and ATP, damaging the cell membrane and increasing its permeability [8].

An application of ozone gas against microorganisms capable of contaminating respirators is reported in the literature. Findings show that ozone achieved high-level disinfection against *P. aeruginosa* without damaging or degrading the filtration capacity of the respirator itself. The conditions of the experiment involved the use of 400 ppm of ozone for two hours at a relative humidity of 80% [42]. Another formulation, which used an atmospheric plasma reactor capable of generating ozone, managed to inactivate *P. aeruginosa* [43].

To our knowledge, no studies on the antibacterial activity of ozone on *S. dysgalacatie* have been reported in the literature.

*E. faecium* and *E. faecalis* are considered among the most important pathogens responsible for infections in hospitals as well as being the most frequent bacteria in chronic wounds [44]. Data on the antimicrobial activity of ozone gel on *E. faecalis* confirm those reported in the literature for other formulations. It was reported that *E. faecalis* is able to penetrate the dentinal tubules and that gaseous ozone, together with other conventional molecules such as NaOCl, can eliminate the bacterium [45]. Other endodontic studies confirmed the action of ozone against *E. faecalis* [46].

Several articles reported the antifungal activity of ozone on *C. albicans*. It has been seen that the effect of ozone ultrafine bubble water leads to a reduced viable count of *C. albicans* in biofilms compared to the control group. However, it was seen that biofilms formed in 24 h were not completely removed [47]. Furthermore, ozone gas acts on antifungal-resistant *C. albicans* biofilms on dentures and urethral catheters. Several studies have been carried out, confirming the thesis that gaseous ozone can help to eliminate biofilms completely; other studies, however, demonstrate that ozone is able to partially reduce the mass of biofilms [48]. Further studies done on *C. albicans* resistant to endodontic treatment demonstrate that gaseous ozone, when applied over varying durations and combined with 2% chlorhexidine, leads to the complete elimination of the fungus [49].

*Candida* mainly affects the oral cavity, and on the basis of this, studies have been carried out on different species of *Candida*: *C. albicans*, *C. glabrata*, and *C. parapsilosis*. Monzillo V. et al. (2020) compared the action of ozone in gel formulation (geliO3) with that of chlorhexedine gel and observed a better antifungal action of ozone. In fact, geliO3 has better characteristics of staying on the wound as it is more hydrophobic than the gel made with chlorhexidine, which is an aqueous gel and, therefore, has a limited stay on the mucous membranes [50]. To our knowledge, no studies on the antifungal activity of ozone on *C. kefir* have been reported in the literature.

Regarding the anti-inflammatory activity of the ozone gel observed in our study, many reports described how ozone has pro-oxygenating effects and stimulates the repair of damaged tissues through the activation of nuclear factor 2 (Nrf2), the main regulator in multiple cytoprotective responses and a key element in a wide field of diseases such as tumors [51] and neurodegenerative processes [52]. The anti-inflammatory activity observed at low doses of ozone is due to an increase in the cell cycle mediated by the synthesis of growth factors and the activation of the NF-κB factor, which triggers transcription of the genes of pro-inflammatory molecules, such as interleukin 8 (IL-8) and tumor necrosis factor α (TNF-α) and TGF-β [11] in infections, allergic dermatitis, skin ulcers, psoriasis, and fibromyalgia.

The ability of ozone to promote wound healing has been attributed to several mechanisms: it leads to an increase in the diameter of capillaries and a consequent increase in oxygen supply [53]; it activates immune system cells, leading to the release of cytokines such as interferon, interleukins, and TNF, as well as the release of factors that stimulate granulocytes [54]; ozone induces the expression of platelet-derived growth factors, endothelial growth factors, and cell cycle D1 proteins [54,55]; moreover, it promotes the local expression of epidermal growth factor while decreasing the expression of TNF-α [53].

Ozonated water tested on zebrafish models demonstrated that, after caudal fin resection, treatment with the solution accelerates tissue regeneration compared to the control group. At 24, 48, and 72 h post-amputation, the length of the caudal fin is significantly greater in the treated group than in the untreated group. Moreover, Hao et al. (2015) investigated cell regeneration in relation to TNF-α levels. It was found that from the time of fin resection up to 24 h ozone induces an increase in TNF-α; however, between 24 and 72 h, there is a decrease in TNF-α levels due to ozone, corresponding to the peak of regenerative activity [53]. In the clinical field, the effectiveness of ozone has been tested for treating diabetic foot ulcers. The administration of gaseous ozone at a concentration of 35 µg/mL in 30 min cycles, repeated over time, has shown improved wound healing; after 1 week, the ozone-treated group displayed a significantly lower wound score compared to the control group, and after 12 weeks, the group treated with ozone therapy had a higher wound healing rate compared to the untreated group: 78% versus 58.3%, respectively. Sun et al. (2024) similarly observed that 1 week after ozone therapy, there was a decrease in TNF-α levels in the treated group compared to the control, with a reduction of 2.85 ng/L [56]. Ozone gel confirmed these results by promoting a greater proliferation of human keratinocytes and fibroblasts, which are key cells involved in tissue repair.

Other evidence confirms that TNF-α levels are higher in subjects with a reduced wound healing capacity, levels that decrease in concomitance with the healing process. Furthermore, in murine models, it has been observed that the inhibition of TNF-α leads to improved collagen deposition, concluding that in the absence of infection, high levels of TNFα hinder wound healing [57].

Finally, the ozone-gel formulation studied in this work exhibits high stability over time. It has been demonstrated that ozone remains stable and active in this formulation for 30 days. This represents a significant advantage compared to other formulations, such as ozonated water-based ones, where ozone concentration is reported to halve every 110 h when stored at 5 °C, and every 9 h when stored at 20 °C [58].

## 5. Conclusions

The ozone formulation in the gel described here has a stability of over 30 months, which facilitates its use compared to formulations that lose efficacy quickly. The ozone gel shows antimicrobial and antibiofilm activity in all ATCC microorganisms examined and on most clinical isolates. Higher concentrations of the ozone gel were also useful in the dispersion of preformed biofilm. The ozone gel also showed anti-inflammatory activity in co-treatment with LPS by reducing the production of TNF-α and regenerative activity on human fibroblasts and keratinocytes. Given all these antimicrobial, anti-inflammatory, and regenerative characteristics, the ozone gel could be, in this formulation, used in the treatment of wounds. A possible application could be the use of a small amount of gel over a sterile gauze compressed on the exposed wound area for a few minutes. After this treatment, the ozone-gel gauze can be removed or replaced until the complete healing. The possibility of self-administration at home without the need for medical personnel is an important benefit. Moreover, the high stability allows the ozone gel to be stored at room temperature for long periods, in non-refrigerated storage conditions commonly available in household environments, without compromising its therapeutic properties.

## Figures and Tables

**Table 1:** Minimal Inhibitory Concentration (MIC) of ozone gel against different ATCC microorganisms.

	MIC (µL/mL)	Positive Control MIC (µL/mL) ^a^
*Staphylococcus aureus* ATCC^®^ 25923 ™	125	1
*Staphylococcus epidermidis* ATCC^®^ 35984 ™	250	0.25
*Pseudomonas aeruginosa* ATCC^®^ PAO-1 ™	250	2
*Escherichia coli* ATCC^®^ 86963 ™	250	1
*Candida albicans* ATCC MYA-2876	62.5	2

MIC was evaluated by standardized CLSI methods. ^a^, the positive control for Gram-positive and Gram-negative bacteria was gentamicin, and that for *Candida albicans* was fluconazole.

## Data Availability

All data used to support the findings of this study are available from the corresponding author upon request.

## Supplementary Materials

The following supporting information can be downloaded at: https://www.mdpi.com/article/10.3390/pharmaceutics16121580/s1. Figure S1. Antibiofilm activity of ozone-gel on *S. aureus* ATCC 25923. MIC was evaluated by broth dilution method. Figure S2. Antibiofilm activity of ozone-gel on *S. epidermidis* ATCC 35984. MIC was evaluated by broth dilution method. Figure S3. Antimicrobial and antibiofilm activity of ozone-gel on *E. coli* ATCC 86963. Figure S4. Antibiofilm activity of ozone-gel on *P. aeruginosa* ATCC PAO-1. Figure S5. Antibiofilm activity of ozone-gel on *C. albicans* CAF2-1. Table S1. Antimicrobial and antibiofilm activity of the ozone-gel on gram negative clinical isolates. Table S2. Antimicrobial and antibiofilm activity of the ozone-gel on gram positive clinical isolates. Table S3. Antimicrobial and antibiofilm activity of the ozone-gel on *Candida* clinical isolates.

## References

[1] Raziyeva K., Kim Y., Zharkinbekov Z., Kassymbek K., Jimi S., Saparov A. (2021). Immunology of Acute and Chronic Wound Healing. Biomolecules.

[2] Sen C.K. (2021). Human Wound and Its Burden: Updated 2020 Compendium of Estimates. Adv. Wound Care.

[3] Rayate A.S., Nagoba B.S., Mumbre S.S., Mavani H.B., Gavkare A.M., Deshpande A.S. (2023). Current Scenario of Traditional Medicines in Management of Diabetic Foot Ulcers: A Review. World J. Diabetes.

[4] Uberoi A., McCready-Vangi A., Grice E.A. (2024). The Wound Microbiota: Microbial Mechanisms of Impaired Wound Healing and Infection. Nat. Rev. Microbiol..

[5] Azzopardi E.A., Azzopardi E., Camilleri L., Villapalos J., Boyce D.E., Dziewulski P., Dickson W.A., Whitaker I.S. (2014). Gram Negative Wound Infection in Hospitalised Adult Burn Patients-Systematic Review and Metanalysis. PLoS ONE.

[6] Sun H., Pulakat L., Anderson D.W. (2020). Challenges and New Therapeutic Approaches in the Management of Chronic Wounds. Curr. Drug Targets.

[7] Huang Q., Ng P.H., Pinheiro Marques A.R., Cheng T.H., Man K.Y., Lim K.Z., MacKinnon B., Huang L., Zhang J., Jahangiri L. (2023). Effect of Ozone Nanobubbles on the Microbial Ecology of Pond Water and Safety for Jade Perch (*Scortum barcoo*). Aquaculture.

[8] Zhang Y.Q., Wu Q.P., Zhang J.M., Yang X.H. (2011). Effects of Ozone on Membrane Permeability and Ultrastructure in Pseudomonas Aeruginosa. J. Appl. Microbiol..

[9] Rangel K., Cabral F.O., Lechuga G.C., Carvalho J.P.R.S., Villas-Bôas M.H.S., Midlej V., De-Simone S.G. (2021). Detrimental Effect of Ozone on Pathogenic Bacteria. Microorganisms.

[10] Bitter K., Vlassakidis A., Niepel M., Hoedke D., Schulze J., Neumann K., Moter A., Noetzel J. (2017). Effects of Diode Laser, Gaseous Ozone, and Medical Dressings on *Enterococcus faecalis* Biofilms in the Root Canal Ex Vivo. BioMed Res. Int..

[11] Fitzpatrick E., Holland O.J., Vanderlelie J.J. (2018). Ozone Therapy for the Treatment of Chronic Wounds: A Systematic Review. Int. Wound J..

[12] Bocci V., Borrelli E., Travagli V., Zanardi I. (2009). The Ozone Paradox: Ozone Is a Strong Oxidant as Well as a Medical Drug. Med. Res. Rev..

[13] Koundle P., Nirmalkar N., Momotko M., Boczkaj G. (2024). Ozone Nanobubble Technology as a Novel AOPs for Pollutants Degradation under High Salinity Conditions. Water Res..

[14] Liu L., Zeng L., Gao L., Zeng J., Lu J. (2022). Ozone Therapy for Skin Diseases: Cellular and Molecular Mechanisms. Int. Wound J..

[15] Zhang Y., Zhang C., Wu B., Li C., Lin J., Huang P. (2023). Thermoresponsive Ozone-Enriched Spray Gel for Postsurgical Treatment of Hepatocellular Carcinoma. ACS Nano.

[16] Khachatryan G., Khachatryan L., Krystyjan M., Lenart-Boroń A., Krzan M., Kulik K., Białecka A., Grabacka M., Nowak N., Khachatryan K. (2022). Preparation of Nano/Microcapsules of Ozonated Olive Oil in Hyaluronan Matrix and Analysis of Physicochemical and Microbiological (Biological) Properties of the Obtained Biocomposite. Int. J. Mol. Sci..

[17] Varghese L.J., Lahiri B., Penumatsa N.V., Soans C.R., Sekar A., Nasyam F.A. (2024). Effectiveness of Topical Ozone Gel Application in the Management of Postextraction Wound Healing: An In Vivo Study. J. Contemp. Dent. Pract..

[18] Colombo M., Gallo S., Garofoli A., Poggio C., Arciola C.R., Scribante A. (2021). Ozone Gel in Chronic Periodontal Disease: A Randomized Clinical Trial on the Anti-Inflammatory Effects of Ozone Application. Biology.

[19] Nakonechna A., Dore P., Dixon T., Khan S., Deacock S., Holding S., Abuzakouk M. (2014). Immediate Hypersensitivity to Chlorhexidine Is Increasingly Recognised in the United Kingdom. Allergol. Immunopathol..

[20] Saini R. (2011). Ozone Therapy in Dentistry: A Strategic Review. J. Nat. Sci. Biol. Med..

[21] Leon B.R., Romary D.J., Landsberger S.A., Bradner K.N., Ramirez M., Lubitz R.M. (2022). Risks of Ozonated Oil and Ozonated Water on Human Skin: A Systematic Review. Int. Wound J..

[22] Porcaro G., Amosso E., Baldoni M. (2019). Treatment of Osteoradionecrosis of the Jaw with Ozone in the Form of Oil-Based Gel: 1-Year Follow-Up. J. Contemp. Dent. Pract..

[23] Bakke R., Kommedal R., Kalvenes S. (2001). Quantification of Biofilm Accumulation by an Optical Approach. J. Microbiol. Methods.

[24] Białoszewski D. (2004). The Impact of Liquid Ozone on the Quality of Surgical Cement Fillings in the Marrow Cavity of Long Bones. Ortop. Traumatol. Rehabil..

[25] Uzun G., Mutluoğlu M., Karagöz H., Memiş A., Karabacak E., Ay H. (2014). Pitfalls of Intralesional Ozone Injection in Diabetic Foot Ulcers: A Case Study. J. Am. Coll. Clin. Wound Spec..

[26] Mielko K.A., Jabłoński S.J., Milczewska J., Sands D., Łukaszewicz M., Młynarz P. (2019). Metabolomic Studies of Pseudomonas Aeruginosa. World J. Microbiol. Biotechnol..

[27] Lakhundi S., Zhang K. (2018). Methicillin-Resistant Staphylococcus Aureus: Molecular Characterization, Evolution, and Epidemiology. Clin. Microbiol. Rev..

[28] Chong K.K.L., Tay W.H., Janela B., Yong A.M.H., Liew T.H., Madden L., Keogh D., Barkham T.M.S., Ginhoux F., Becker D.L. (2017). Enterococcus Faecalis Modulates Immune Activation and Slows Healing During Wound Infection. J. Infect. Dis..

[29] Silva S., Negri M., Henriques M., Oliveira R., Williams D.W., Azeredo J. (2012). Candida Glabrata, Candida Parapsilosis and Candida Tropicalis: Biology, Epidemiology, Pathogenicity and Antifungal Resistance. FEMS Microbiol. Rev..

[30] Okuno E., Jarros I.C., Bonfim-Mendonça P.S., Vicente de Rezende G., Negri M., Svidzinski T.E. (2018). Candida Parapsilosis Isolates from Burn Wounds Can Penetrate an Acellular Dermal Matrix. Microb. Pathog..

[31] Chiang S.M., Schellhorn H.E. (2012). Regulators of Oxidative Stress Response Genes in *Escherichia coli* and Their Functional Conservation in Bacteria. Arch. Biochem. Biophys..

[32] Davies M.J. (2004). Reactive Species Formed on Proteins Exposed to Singlet Oxygen. Photochem. Photobiol. Sci..

[33] Stadtman E.R. (2006). Protein Oxidation and Aging. Free Radic. Res..

[34] Nyström T. (2002). Translational Fidelity, Protein Oxidation, and Senescence: Lessons from Bacteria. Ageing Res. Rev..

[35] Bialoszewski D., Bocian E., Bukowska B., Czajkowska M., Sokol-Leszczynska B., Tyski S. (2010). Antimicrobial Activity of Ozonated Water. Med. Sci. Monit..

[36] Zeng J., Dou J., Gao L., Xiang Y., Huang J., Ding S., Chen J., Zeng Q., Luo Z., Tan W. (2020). Topical Ozone Therapy Restores Microbiome Diversity in Atopic Dermatitis. Int. Immunopharmacol..

[37] Roth A., Elkashif A., Selvamani V., Stucky R.A., Seleem M.N., Ziaie B., Rahimi R. (2020). Wearable and Flexible Ozone Generating System for Treatment of Infected Dermal Wounds. Front. Bioeng. Biotechnol..

[38] Prebeg D., Katunarić M., Budimir A., Pavelić B., Šegović S., Anić I. (2016). Antimicrobial Effect of Ozone Made by KP Syringe of High-Frequency Ozone Generator. Acta Stomatol. Croat..

[39] Baghal Asghari F., Dehghani M.H., Dehghanzadeh R., Farajzadeh D., Shanehbandi D., Mahvi A.H., Yaghmaeian K., Rajabi A. (2021). Performance Evaluation of Ozonation for Removal of Antibiotic-Resistant Escherichia Coli and Pseudomonas Aeruginosa and Genes from Hospital Wastewater. Sci. Rep..

[40] Breidablik H.J., Lysebo D.E., Johannessen L., Skare Å., Andersen J.R., Kleiven O. (2020). Effects of Hand Disinfection with Alcohol Hand Rub, Ozonized Water, or Soap and Water: Time for Reconsideration?. J. Hosp. Infect..

[41] Szeto W., Yam W.C., Huang H., Leung D.Y.C. (2020). The Efficacy of Vacuum-Ultraviolet Light Disinfection of Some Common Environmental Pathogens. BMC Infect. Dis..

[42] Manning E.P., Stephens M.D., Dufresne S., Silver B., Gerbarg P., Gerbarg Z., Dela Cruz C.S., Sharma L. (2021). Disinfection of Pseudomonas Aeruginosa from N95 Respirators with Ozone: A Pilot Study. BMJ Open Respir. Res..

[43] Choudhury B., Portugal S., Mastanaiah N., Johnson J.A., Roy S. (2018). Inactivation of Pseudomonas Aeruginosa and Methicillin-Resistant Staphylococcus Aureus in an Open Water System with Ozone Generated by a Compact, Atmospheric DBD Plasma Reactor. Sci. Rep..

[44] Melo L.D.R., Ferreira R., Costa A.R., Oliveira H., Azeredo J. (2021). Author Correction: Efficacy and Safety Assessment of Two Enterococci Phages in an in Vitro Biofilm Wound Model. Sci. Rep..

[45] Tuncay Ö., Er Ö., Demirbuga S., Zorba Y.O., Topçuoğlu H.S. (2016). Effect of Gaseous Ozone and Light-Activated Disinfection on the Surface Hardness of Resin-Based Root Canal Sealers. Scanning.

[46] Hubbezoglu I., Zan R., Tunc T., Sumer Z. (2014). Antibacterial Efficacy of Aqueous Ozone in Root Canals Infected by Enterococcus Faecalis. Jundishapur J. Microbiol..

[47] Shichiri-Negoro Y., Tsutsumi-Arai C., Arai Y., Satomura K., Arakawa S., Wakabayashi N. (2021). Ozone Ultrafine Bubble Water Inhibits the Early Formation of Candida Albicans Biofilms. PLoS ONE.

[48] Zargaran M., Fatahinia M., Zarei Mahmoudabadi A. (2017). The Efficacy of Gaseous Ozone against Different Forms of Candida Albicans. Curr. Med. Mycol..

[49] Noites R., Pina-Vaz C., Rocha R., Carvalho M.F., Gonçalves A., Pina-vaz I. (2014). Synergistic Antimicrobial Action of Chlorhexidine and Ozone in Endodontic Treatment. Biomed. Res. Int..

[50] Monzillo V., Lallitto F., Russo A., Poggio C., Scribante A., Arciola C.R., Bertuccio F.R., Colombo M. (2020). Ozonized Gel Against Four Candida Species: A Pilot Study and Clinical Perspectives. Materials.

[51] Simonetti V., Quagliariello V., Franzini M., Iaffaioli R.V., Maurea N., Valdenassi L. (2019). Ozone Exerts Cytoprotective and Anti-Inflammatory Effects in Cardiomyocytes and Skin Fibroblasts after Incubation with Doxorubicin. Evid. Based Complement. Altern. Med..

[52] Scassellati C., Galoforo A.C., Bonvicini C., Esposito C., Ricevuti G. (2020). Ozone: A Natural Bioactive Molecule with Antioxidant Property as Potential New Strategy in Aging and in Neurodegenerative Disorders. Ageing Res. Rev..

[53] Hao K., Li Y., Feng J., Zhang W., Zhang Y., Ma N., Zeng Q., Pang H., Wang C., Xiao L. (2015). Ozone Promotes Regeneration by Regulating the Inflammatory Response in Zebrafish. Int. Immunopharmacol..

[54] Araneda S., Commin L., Atlagich M., Kitahama K., Parraguez V.H., Pequignot J.-M., Dalmaz Y. (2008). VEGF Overexpression in the Astroglial Cells of Rat Brainstem Following Ozone Exposure. Neurotoxicology.

[55] Valacchi G., Lim Y., Belmonte G., Miracco C., Zanardi I., Bocci V., Travagli V. (2011). Ozonated Sesame Oil Enhances Cutaneous Wound Healing in SKH1 Mice. Wound Repair Regen..

[56] Sun H., Heng H., Liu X., Geng H., Liang J. (2024). Evaluation of the Healing Potential of Short-Term Ozone Therapy for the Treatment of Diabetic Foot Ulcers. Front. Endocrinol..

[57] Ashcroft G.S., Jeong M.-J., Ashworth J.J., Hardman M., Jin W., Moutsopoulos N., Wild T., McCartney-Francis N., Sim D., McGrady G. (2012). TNFα Is a Therapeutic Target for Impaired Cutaneous Wound Healing. Wound Repair Regen..

[58] Bocci V. (2010). Preparation of Ozonated Water and Oil for the Topical Therapy—Ozone as a Drinking Water Disinfectant: Ozone Disinfection to Prevent Nosocomial Infections. OZONE.

