# Supplementary material for: Antibiofilm, Anti-Inflammatory, and Regenerative Properties of a New Stable Ozone-Gel Formulation
Source: Pharmaceutics. 2024 Dec 11;16(12):1580. doi: 10.3390/pharmaceutics16121580 (PMC11676070; doi:10.3390/pharmaceutics16121580)
Supplement: Supplementary file 1 [file pharmaceutics-16-01580-s001.zip › pharmaceutics-3287705-supplementary.pdf]

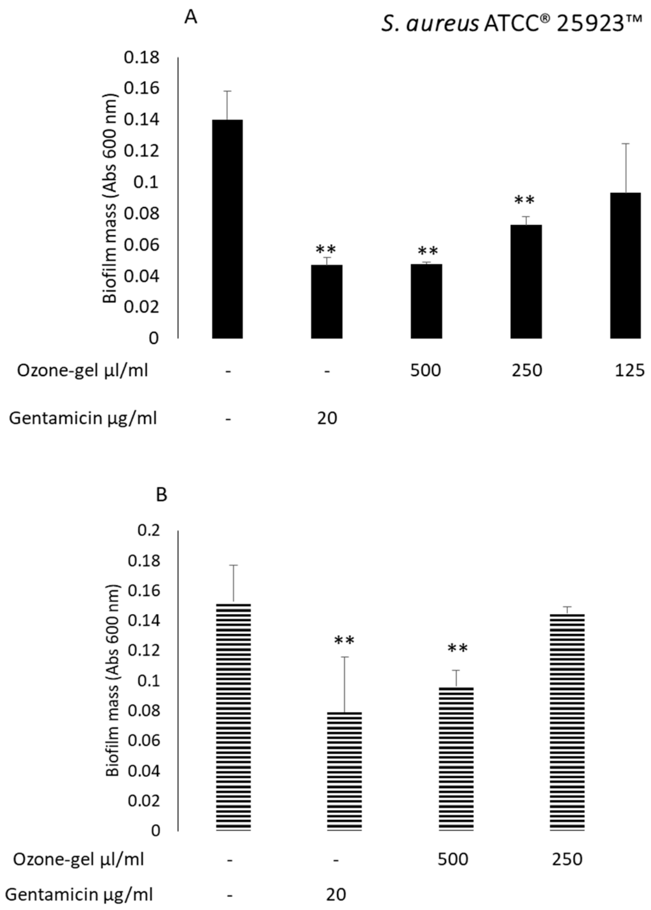

Figure S1. Antibiofilm activity of ozone-gel on *S. aureus* ATCC 25923. MIC was evaluated by broth dilution method. The mass of the biofilm was evaluated by spectrophotometric reading of the bacterial suspension recovered from the biofilm after formation (A) or dispersion (B). Data represent the mean±SD of three independent experiments. **P<0.01, biofilms treated with gentamicin or ozone-gel vs untreated biofilms.

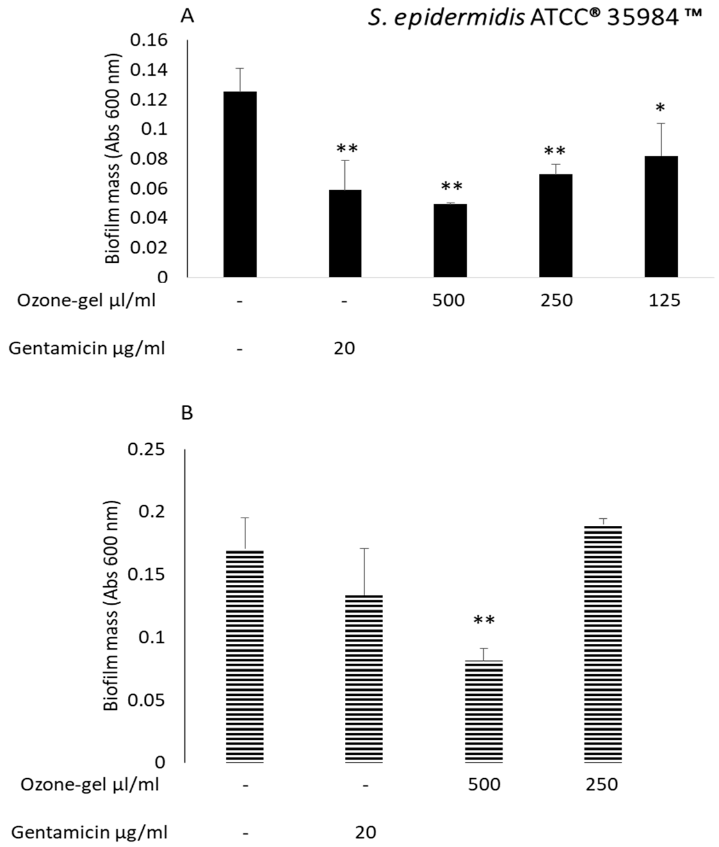

Figure S2. Antibiofilm activity of ozone-gel on *S. epidermidis* ATCC 35984. MIC was evaluated by broth dilution method. The mass of the biofilm was evaluated by spectrophotometric reading of the bacterial suspension recovered from the biofilm after formation (A) or dispersion (B). Data represent the mean±SD of three independent experiments. * P<0.05, **P<0.01, biofilms treated with gentamicin or ozone-gel vs untreated biofilms.

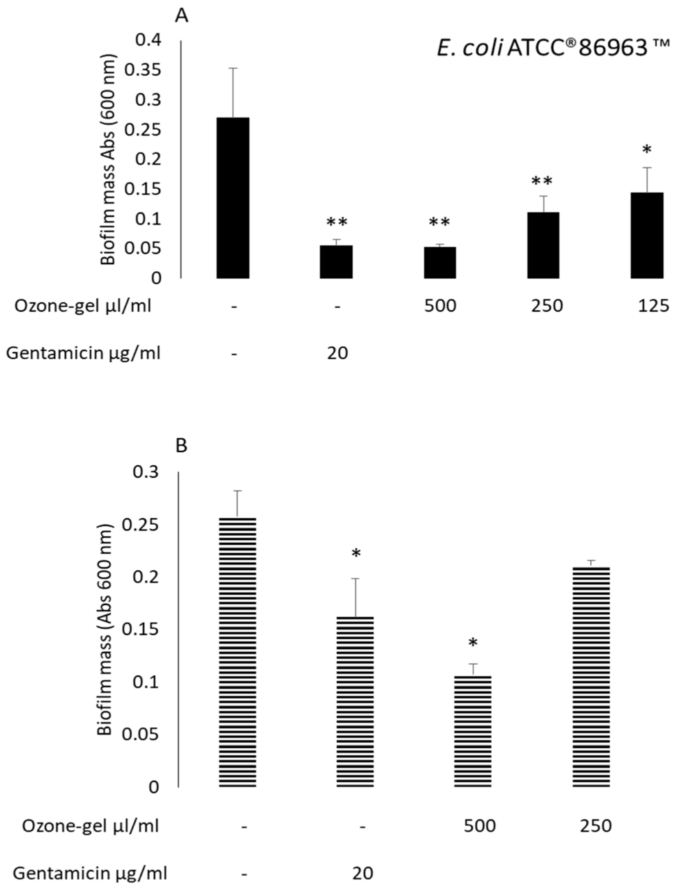

Figure S3. Antimicrobial and antibiofilm activity of ozone-gel on E. coli ATCC 86963.

MIC was evaluated by broth dilution method. The mass of the biofilm was evaluated by spectrophotometric reading of the bacterial suspension recovered from the biofilm after formation (A) or dispersion (B). Data represent the mean±SD of three different repetitions.

* $P<0.05$, ** $P<0.01$, biofilms treated with gentamicin or ozone-gel vs untreated biofilms

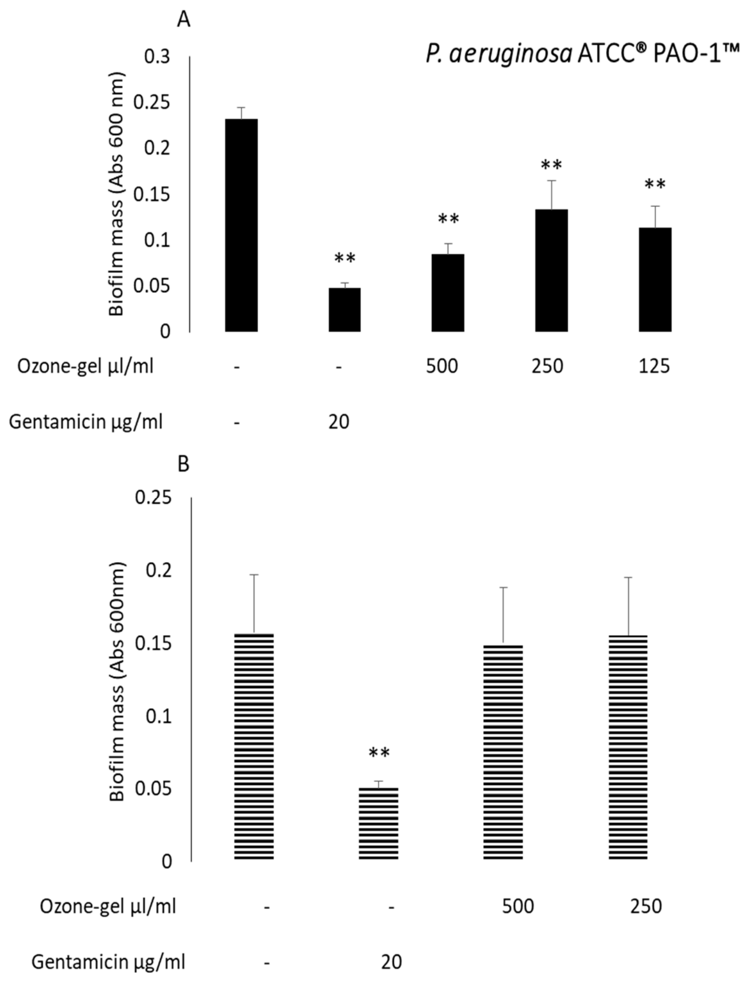

Figure S4. Antibiofilm activity of ozone-gel on *P. aeruginosa* ATCC PAO-1. MIC was evaluated by broth dilution method. The mass of the biofilm was evaluated by spectrophotometric reading of the bacterial suspension recovered from the biofilm after formation (A) or dispersion (B). Data represent the mean±SD of three independent experiments. **P<0.01, biofilms treated with gentamicin or ozone-gel vs untreated biofilms

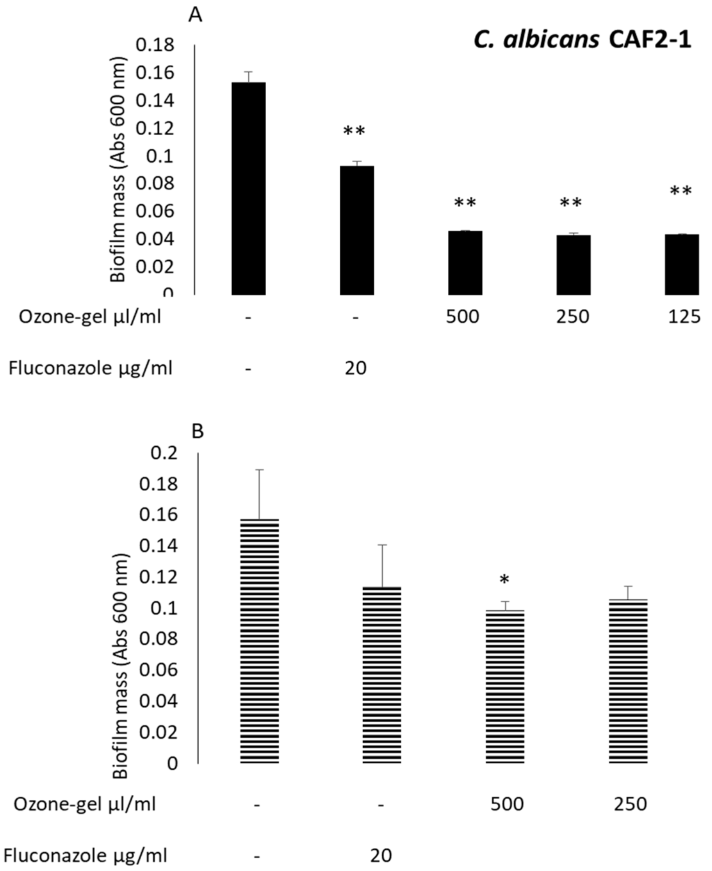

Figure S5. Antibiofilm activity of ozone-gel on *C. albicans* CAF2-1. MIC was evaluated by broth dilution method. The mass of the biofilm was evaluated by spectrophotometric reading of the yeast suspension recovered from the biofilm after formation (A) or dispersion (B). Data represent the mean±SD of three independent experiment. * P<0.05, **P<0.01, biofilms treated with fluconazole or ozone-gel vs untreated biofilms.

Table S1. Antimicrobial and antibiofilm activity of the ozone-gel on gram negative clinical isolates

	Clinical Isolates	Drug Resistance	MIC	% Biofilm formation inhibition			% Biofilm dispersion	
				250 µl/ml	125 µl/ml	62.5 µl/ml	250 µl/ml	125 µl/ml
Escherichia coli	UR20191956	-	250 µl/ml	81±1.5**	75±3.3**	36±1.6*	36±5.1**	NT
	UR20192300	-	250 µl/ml	62±3.4**	53±7.1**	12±14.5	34±21.4	NT
	UR20192302	-	250 µl/ml	75±1.3*	69±3.6*	27±17.1	51±5.1*	NT
	UR20191986	ESBL	125 µl/ml	69±0.7*	72±1.0*	0	32±7.4*	NT
	UR20191914	ESBL	125 µl/ml	76±2.3**	75±0.9*	52±11.8	70±12.1*	NT
	VA30067823	ESBL	125 µl/ml	60±0.9**	71±1.5**	0	73±26.3*	NT
Klebsiella pneumoniae	VA30067456	KPC	125 µl/ml	77±2.1**	81±0.4**	6±27.3	0	0
	VA30067596	KPC	125 µl/ml	76±1,3*	76±0.5*	49±6.1	62±14.7*	58±12*
	VA30067145	KPC	125 µl/ml	72±2.5*	75±4.4*	0	57±4.9**	49±21*
	VA30067241	KPC	125 µl/ml	70±1**	64±0.8*	39±49.4	46±4.2*	45±23.7
	RE10042905	KPC	125 µl/ml	86±0.5**	83±0.8**	31±24.2	81±1.4**	58±41.7
	UR20191855	KPC	125 µl/ml	83±2.5**	78±2**	13±17.6	50±2.2*	37±37.5
	VA30067674	KPC	125 µl/ml	88±0.5**	89±0.6**	64±7.9	9±15.3	30±21.4
Pseudomonas aeruginosa	UR20191959	-	250 µl/ml	78±17.9*	80±0.5*	36±40.7**	0	0
	UR20191909	-	250 µl/ml	70±13.2**	51±10.5**	26±20.7**	0	2±12
	UR20192320	-	250 µl/ml	56±24.8	21±16.3	27±33.9**	1±45	0
	VA30067674	-	250 µl/ml	55±6.4	2±6.3	0	6±37	0
	RE10043262	-	250 µl/ml	82±2.7**	71±3.9**	26±40.8**	12±38	0
	VA30067673	-	250 µl/ml	37±27.2	41±10.6	0	22±35	0

The MIC was evaluated by dilution method in broth. The mass of the biofilm was evaluated by spectrophotometric reading of the bacterial suspension recovered from the biofilm after formation or dispersion. The data represent the mean±SD of three individual experiments. **P<0.01, *P<0.05, biofilm treated with ozone-gel *vs* untreated biofilms.

Table S2. Antimicrobial and antibiofilm activity of the ozone-gel on gram positive clinical isolates

	Isolates	MDRO	MIC	% Biofilm formation inhibition			% Biofilm dispersion	
				250 µl/ml	125 µl/ml	62.5 µl/ml	250 µl/ml	125 µl/ml
Staphylococcus aureus	VA30067711	MRSA	125 µl/ml	82±1.6**	80±1.9**	17±23.3	63±8.4**	6±7.6
	RE10043262	MRSA	125 µl/ml	80±1.8**	72±8.0**	0	57±7.8*	16±5.4
	VA30067951	MRSA	125 µl/ml	72±5.5*	39±7.8	0	63±4.3*	0
	VA30067747	MRSA	125 µl/ml	82±2.2*	77±1.5*	9±12.1	57±6.0	24±4.0
	VA30067717	MRSA	125 µl/ml	82±0.3**	78±4.0**	44±5.8*	60±6.3	23±30.1
	VA30067941	MRSA	250 µl/ml	73±5.9**	47±13.0*	0	41±22.9	0
	VA30067938	MRSA	125 µl/ml	90±0.3**	82±2.4**	45±21.8*	52±10.3**	22±11.8
	VA30067972	MRSA	250 µl/ml	73±2.3*	29±8.0	0	4±17.3	0
	VA30067960	MRSA	125 µl/ml	81±1.5**	76±2.2**	0	33±22.8	32±5.0
	VA3006742	MRSA	125 µl/ml	86±1.4**	77±15.0*	9±6.2	51±2.3*	22±19.2
	EM00145410	MRSA	250 µl/ml	88±0.4*	79±4.4*	34±8.4	43±14.7	28±15.2
	EM00145458	MRSA	250 µl/ml	87±0.5**	82±2.0*	43±3.2	50±5.9**	29±13.2
	RE10043376	MRSA	125 µl/ml	86±0.4**	82±2.2*	39±7.0	51±6.7*	2±24.3
	UR20192789	MRSA	125 µl/ml	83±1.8**	76±4.3*	0	23±11.3	0
	UR20192680	MRSA	125 µl/ml	81±0.7**	76±4.0**	17±21.8	5±21.9	0
Streptococccus. dysgalactiae	EM00145068	-	125 µl/ml	81±1.1*	85±1.0*	58±17.6	38±19.6	18±25.9
	GE80071664	-	125 µl/ml	77±9.7	64±9.6	59±6.1	90±6.2**	89±4.6**
	GE80071657	-	125 µl/ml	75±19.2	73±18.8	72±5.7	60±7.5*	55±7.3*
Enterococcus faecalis	UR20191898	-	125 µl/ml	62±10.5**	38±9.4**	0	36±5.5*	4±12.6
	UR20191805	-	125 µl/ml	50±2.0*	20±16.2	0	17±3.2	0
	UR20191974	-	250 µl/ml	43±4.0*	37±4.4*	13±18.1	26±8.0*	0
Enterococcus faecium	VA30067684	-	125 µl/ml	64±2.8*	10±12.9	4±1.0	7±19.2	0
	VA30067702	-	125 µl/ml	68±1.6**	0	0	8±10.4	0
	VA30067728	-	125 µl/ml	58±3.9**	19±11.6	0	7±29.6	0
	VA30067767	VanA	125 µl/ml	69±3.7**	29±11.5*	0	0	0
	VA30067786	VanA	125 µl/ml	73±1.3**	30±13.9*	0	0	0
	VA30067786	VanA	125 µl/ml	74±1.5**	37±17.5*	19±12.7	21±13.9	0

The MIC was evaluated by dilution method in broth. The mass of the biofilm was evaluated by spectrophotometric reading of the bacterial suspension recovered from the biofilm after formation or dispersion. The data represent the mean±SD of three individual experiments. **P<0.01, *P<0.05, biofilm treated or ozone-gel *vs* untreated biofilms.

Table S3. Antimicrobial and antibiofilm activity of the ozone-gel on *Candida* clinical isolates

	Isolates	MIC	% Biofilm formation inhibition			% Biofilm dispersion	
			250 μl/ml	125 μl/ml	62.5 μl/ml	250 μl/ml	125 μl/ml
Candida albicans	UR20198838	62.5 μl/ml	90±1.5**	90±2.2**	92±0.6**	35 ±46.3	45 ±27.5
	UR20198903	125 μl/ml	71±2.9**	46±10.4*	33±34.5	30 ±17.6	42 ±10.4
	MI60031844	62.5 μl/ml	75±1.9**	73±1.7**	64±6.0**	74 ±10.8**	41 ±11.9*
	MI60031856	62.5 μl/ml	92±3.0**	85±13.0**	93±0.6**	68 ±10.2**	45 ±16.5*
Candida glabrata	UR20191985	62.5 μl/ml	52±0.6**	55±1.4**	49±5.6**	71±1.8	47±23.3
	UR20192313	62.5 μl/ml	87±0.1**	84±4.0**	81±4.9**	46±14.0	2±27.1
	GE80069747	125 μl/ml	52±2.2*	56±1.7*	581.1*	39±11.2*	7±49.6
	GE80069750	62.5 μl/ml	63±0.7**	64±1.2**	63±0.7**	41±9.3	49±11.4
	VA30070584	62.5 μl/ml	73±18.1**	34±1.6**	43±13.3*	72±1.6	58 ±1.7
	UR20198745	125 μl/ml	83±7.7*	78±1.1*	79±1.1*	48 ±5.7	38 ±4.2
Candida kefir	UR20192097	62.5 μl/ml	74±0.2*	76±0.6*	74±2.6*	81±2.3**	59±10.4*
Candida parapsilosis	EM00144852	125 μl/ml	73±3.1**	68±7.9**	12±7.1	74±4.7**	59±3.4**

The MIC was evaluated by dilution method in broth. The mass of the biofilm was evaluated by spectrophotometric reading of the bacterial suspension recovered from the biofilm after formation or dispersion. The data represent the mean±SD of three individual experiments. **P<0.01, *P<0.05, biofilm treated with ozone-gel *vs* untreated biofilms.
